# Sex-Based Comparative Analysis of Outcomes Following Minimally Invasive Direct Coronary Artery Bypass: A 20-Year Study

**DOI:** 10.3390/jcdd12120460

**Published:** 2025-11-27

**Authors:** Maria Comanici, Abu A. Farmidi, Fabio De Robertis, Nandor Marczin, Sunil K. Bhudia, Toufan Bahrami, Shahzad G. Raja

**Affiliations:** 1 Department of Cardiac Surgery, Harefield Hospital, London UB9 6JH, UK; maria.comanici1@nhs.net (M.C.); abu.farmidi@nhs.net (A.A.F.); fabio.derobertis1@nhs.net (F.D.R.); s.bhudia@nhs.net (S.K.B.); t.bahrami@nhs.net (T.B.); 2 Department of Anaesthesia and Critical Care, Harefield Hospital, London UB9 6JH, UK; nandor.marczin@nhs.net; 3 Faculty of Medicine, Department of Surgery and Cancer, Imperial College, London SW7 2AZ, UK

**Keywords:** female, MIDCAB, outcomes, sex, survival

## Abstract

Background: Despite the increasing adoption of minimally invasive direct coronary artery bypass (MIDCAB), data on its long-term outcomes—particularly regarding sex-based differences—remain limited. This study presents a robust 20-year analysis comparing males and females, assessing perioperative outcomes, long-term survival, and independent predictors of mortality to inform sex-sensitive clinical decision-making. Methods: A retrospective cohort analysis of 676 patients (138 females, 538 males) undergoing MIDCAB was performed. Propensity score matching (PSM) generated balanced female and male cohorts (*n* = 129 each). Preoperative demographics, short-term outcomes, and long-term survival were assessed using Kaplan–Meier analysis and Cox regression modelling. Results: In unmatched cohorts, females exhibited significantly lower NYHA class distribution (*p* = 0.011) and higher atrial fibrillation prevalence (*p* = 0.038), with otherwise comparable comorbidities. Propensity score matching achieved cohort balance, and short-term outcomes—including 30-day mortality, stroke/TIA, and reoperation—were similar across sexes. Kaplan–Meier analysis of matched cohorts revealed no significant survival difference (log-rank *p* = 0.3370), though females demonstrated greater 20-year survival than males (77.6% versus 55.8%). In females, age 70–79 (HR 2.66; 95% CI: 1.02–6.95; *p* = 0.046) and cerebrovascular disease (HR 5.33; 95% CI: 1.49–19.03; *p* = 0.010) were independently associated with mortality. In males, significant predictors included diabetes (HR 1.86; 95% CI: 1.02–3.38; *p* = 0.042), chronic kidney disease (HR 4.92; 95% CI: 1.21–20.02; *p* = 0.026), pulmonary disease (HR 2.35; 95% CI: 1.20–4.60; *p* = 0.013), cerebrovascular disease (HR 4.77; 95% CI: 1.97–11.56; *p* < 0.001), and reduced left ventricular ejection fraction (HR 0.17; 95% CI: 0.06–0.43; *p* < 0.001). Conclusions: This 20-year study, the longest to date, demonstrates that MIDCAB achieves durable and equivalent long-term survival in males and females. It highlights sex-specific predictors of mortality, emphasizing the necessity for personalized preoperative risk assessment and postoperative management.

## 1. Introduction

Cardiovascular disease remains the leading cause of mortality across Europe, accounting for 40% of deaths in men and 49% in women [1]. Sex-specific differences in presentation, diagnosis, and management of ischemic heart disease have been increasingly recognized [2]. Women undergoing coronary artery bypass grafting (CABG) tend to be older, present with more comorbidities, and experience higher rates of perioperative complications, including mortality [3,4]. Whether female sex constitutes an independent risk factor after CABG remains debated [5,6].

In patients with isolated left anterior descending (LAD) artery stenosis who are unsuitable for percutaneous coronary intervention, minimally invasive direct coronary artery bypass (MIDCAB) offers a sternal-sparing, off-pump approach using the left internal mammary artery (LIMA) [7]. Compared to conventional sternotomy-based CABG, MIDCAB reduces surgical trauma, shortens recovery time, and avoids cardiopulmonary bypass—particularly advantageous in women given their elevated risk of sternal wound complications and transfusion requirements [8,9]. However, female anatomy can pose challenges during MIDCAB, including breast tissue management, smaller thoracic dimensions, and higher rates of postoperative wound healing issues [10].

While MIDCAB has demonstrated excellent short- and mid-term outcomes, long-term data beyond ten years—especially stratified by sex—remain sparse [11,12]. Emerging matched cohort studies suggest that outcomes in women may be equivalent or even superior to men when adjusting for baseline risk factors, but these findings have yet to be validated over extended follow-up [13,14]. Additionally, few investigations have explored sex-specific predictors of late mortality such as insulin-dependent diabetes mellitus (IDDM), atrial fibrillation, and ICU stay duration [15,16].

The primary aim of this study was to assess short-term outcomes and long-term survival in patients undergoing MIDCAB for LAD disease, stratified by sex. The secondary aim was to identify independent, sex-specific predictors of long-term mortality. This work seeks to address the knowledge gap in sex-specific outcomes after MIDCAB and to inform personalized decision-making in coronary revascularization.

## 2. Methods

### 2.1. Study Design and Patient Selection

This retrospective study used prospectively recorded data from cardiac surgeries performed at our institution between February 1996 and September 2023. Ethical approval was granted by the local audit board (QS/SR-MCAB 04/2024), and the need for individual patient consent was waived in accordance with the Declaration of Helsinki.

Patient information was sourced from the institutional Patients Analysis & Tracking System (Dendrite Clinical Systems, Oxford, UK), which is routinely submitted to the National Adult Cardiac Surgery Audit overseen by the National Institute for Cardiovascular Outcomes Research (NICOR). Data refinement involved the removal of duplicates, exclusion of nonadult surgical procedures, resolution of inconsistencies, and correction of clinically implausible values. Variable definitions and database architecture are publicly accessible via NICOR.

The study population included patients who underwent isolated LAD revascularization via MIDCAB. Indications for surgery included LAD lesions unsuitable for percutaneous coronary intervention due to ostial involvement, anatomical complexity, occlusion, or restenosis following stenting. Additional cases involved patients who underwent prior angioplasty of non-LAD vessels and required LAD revascularization, as well as elective hybrid revascularization cases combining MIDCAB and PCI.

### 2.2. Preoperative Variables

A comprehensive set of baseline variables was examined, including patient age, New York Heart Association classification, history of congestive heart failure, previous myocardial infarctions, and prior cardiac surgeries. Cardiovascular risk profiles incorporated the presence of diabetes mellitus, hypercholesterolemia, hypertension, smoking status, chronic renal failure, chronic obstructive pulmonary disease (COPD) or asthma, cerebrovascular disease, peripheral vascular disease, and atrial fibrillation. Coronary anatomy was assessed by extent of disease, left main stem involvement, and left ventricular ejection fraction (LVEF). Operative risk stratification was performed using the logistic EuroSCORE model.

### 2.3. Outcomes Assessed

Short-term outcomes included reoperation for bleeding, tracheostomy, transient ischemic attack or cerebrovascular accident (TIA/CVA), initiation of renal replacement therapy, and all-cause mortality within 30 days of surgery. Long-term outcome analysis focused exclusively on all-cause mortality. This endpoint was selected for its robustness and independence from subjective adjudication, thereby minimizing classification bias. Mortality data were triangulated using both the institutional registry and the National Health Service Spine database. Complete information on postoperative complications and survival status was available for the entire cohort.

### 2.4. Statistical Analysis

Descriptive statistics were applied to continuous and categorical variables according to distribution type. The Lilliefors test was used to evaluate normality. Normally distributed continuous variables were expressed as mean ± standard deviation and compared using the unpaired Student’s *t*-test, while non-normally distributed variables were reported as median with interquartile range and compared using the Mann–Whitney U-test. Categorical data were reported as counts and percentages, with group differences assessed using Pearson’s chi-squared test or Fisher’s exact test where appropriate.

Survival analysis was conducted using Kaplan–Meier estimates with log-rank testing. Curves were annotated with 95% confidence intervals, and survival probabilities along with standard errors were calculated at 1, 5, 10, 15, and 20 years. Prognostic variables associated with long-term mortality were identified using univariable and multivariable Cox proportional hazards modeling. These analyses were performed on the unmatched cohort to preserve sample size and modeling power within each sex group. Statistical significance was defined as a two-sided *p*-value less than 0.05.

To adjust for baseline differences between male and female patients, propensity score matching (PSM) was employed using a logistic regression framework that incorporated all preoperative variables. Each male patient was matched to a female patient using a 1:1 greedy matching algorithm with a caliper width of 0.20 standard deviations of the logit of the propensity score. This caliper size was selected based on its balance between bias reduction and retention of an adequate matched sample size. Narrower calipers risk excluding too many patients, while wider calipers increase the potential for imbalance. Covariate balance before and after matching was assessed using absolute standardized mean differences (ASMD), with values < 0.1 considered indicative of adequate balance. ASMDs were reported for all baseline variables to complement *p* values and provide a more robust assessment of matching quality.

Clinical records were collated in Microsoft Excel (Microsoft Corp., Redmond, WA, USA). All statistical computations were performed using SPSS Statistics software (version 29.0.2.0; IBM Corp., Armonk, NY, USA).

## 3. Results

### 3.1. Demographic Data

The study cohort comprised 676 patients who underwent MIDCAB for LAD revascularization, of which 138 were women and 538 were men. Following 1:1 propensity score matching, 129 male-female pairs were generated for analysis. In the unmatched cohort, women were slightly older than men (64.6 ± 10.7 vs. 63.1 ± 10.5 years; *p* = 0.122), with no significant age difference post-matching (64.1 ± 10.5 vs. 63.8 ± 9.6 years; *p* = 0.562). NYHA class distribution differed significantly between sexes prior to matching (*p* = 0.011) but was balanced thereafter (*p* = 0.814). Cerebrovascular disease and atrial fibrillation were more prevalent in women before matching (6.5% vs. 3.0%, *p* = 0.049; and 0.7% vs. 4.5%, *p* = 0.038, respectively), though differences were neutralized in the matched cohort (Table 1).

Other comorbidities, including diabetes, hypertension, hypercholesterolemia, COPD/asthma, and chronic renal failure, were comparable between sexes in both unmatched and matched groups. Coronary disease extent and LVEF were well distributed across both sexes, with no significant intergroup variation (Table 1).

Logistic EuroSCORE was 3.3 ± 3.1 in women and 3.5 ± 6.5 in men (*p* = 0.490) in the unmatched cohort, and 3.2 ± 3.1 vs. 3.2 ± 4.4 (*p* = 0.974) in the matched cohort. Although female sex is traditionally associated with higher logistic EuroSCORE values, the slightly lower mean score observed in women within this cohort likely reflects the procedural selection and comparable comorbidity burden, with no statistically significant difference between sexes.

### 3.2. Perioperative Outcomes

Perioperative events occurred infrequently and were similar between groups. Reoperation for bleeding was required in 2.9% of women and 2.4% of men (*p* = 0.789); in the matched cohort, these rates remained low (3.1% vs. 2.3%, *p* = 0.702). No tracheostomies were performed in female patients, while one male required the intervention in the unmatched group. Rates of TIA/CVA, renal replacement therapy, and 30-day mortality showed no significant differences pre- or post-matching. Notably, 30-day mortality was 0% in women and 1.1% in men (*p* = 0.213), decreasing further in matched pairs (0% vs. 0.8%; *p* = 0.316). Mean follow-up duration was 10.9 ± 7.4 years in women and 10.5 ± 7.2 years in men in the unmatched cohort (Table 2).

### 3.3. Survival Outcomes

**Figure 1:**
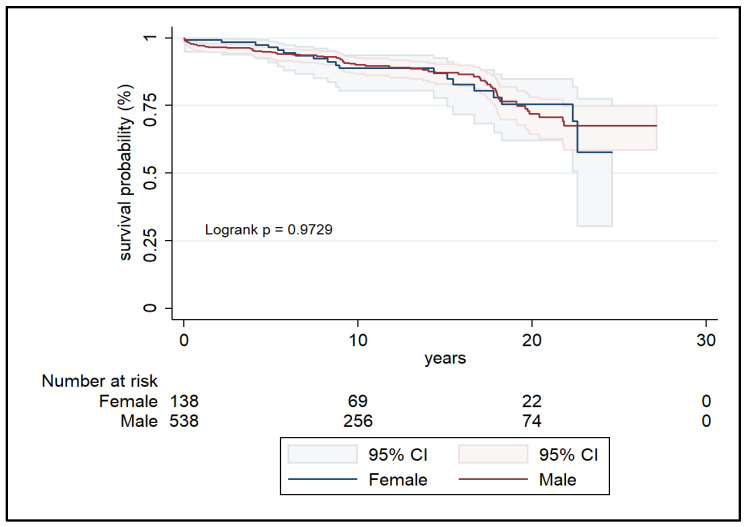
Kaplan–Meier survival curves of unmatched cohorts.

**Figure 2:**
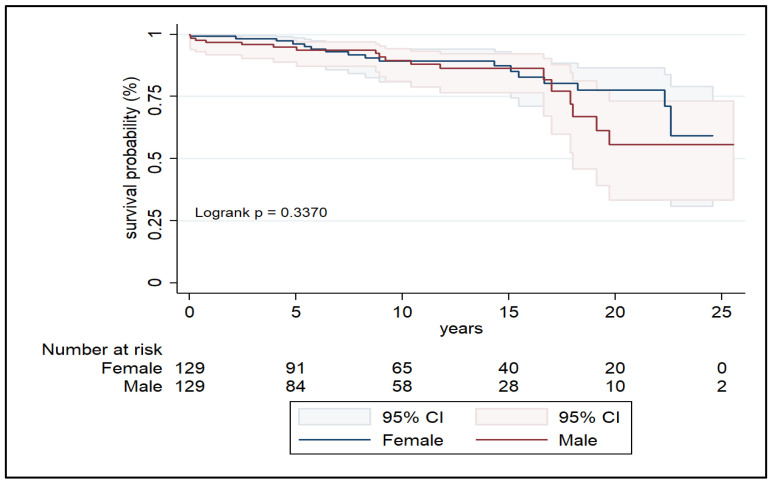
Kaplan–Meier survival curves of matched cohorts.

Kaplan–Meier analysis revealed no significant difference in survival probability between females and males in the unmatched cohort (Figure 1). At 1, 5, 10, 15, and 20 years, survival rates in women were 99.3%, 96.5%, 88.8%, 87.0%, and 75.6%, respectively, compared to 97.2%, 94.9%, 90.1%, 87.2%, and 72.0% in men (Table 3). In the matched cohort (Figure 2), survival remained statistically similar (log-rank *p* = 0.337), with female survival at 20 years reaching 77.6% versus 55.8% in men (Table 4).

### 3.4. Predictors of Long-Term Mortality

Multivariable Cox regression analysis stratified by sex revealed age ≥ 70 years as a significant predictor of late mortality in women (HR 2.66, 95% CI 1.02–6.95; *p* = 0.046). Cerebrovascular disease was also a strong independent predictor (HR 5.33, 95% CI 1.49–19.03; *p* = 0.010) (Table 5).

In men, significant predictors included age *p* = 0.041), prior cardiac surgery (HR 4.51, *p* = 0.008), diabetes (HR 1.86, *p* = 0.042), chronic kidney disease (HR 4.92, *p* = 0.026), pulmonary disease (HR 2.35, *p* = 0.013), and cerebrovascular disease (HR 4.77, *p* < 0.001). Good LVEF was associated with lower mortality (HR 0.17, *p* < 0.001) (Table 6).

## 4. Discussion

This propensity-matched cohort study evaluated long-term outcomes following MIDCAB for isolated LAD artery disease, with specific attention to sex-based differences. The principal findings indicate excellent long-term survival in both male and female patients, with matched women demonstrating a trend toward improved 20-year survival. Perioperative complication rates and 30-day mortality were low and comparable across sexes. Moreover, sex-specific predictors of late mortality were identified, suggesting that clinical risk factors—rather than sex alone—may be more influential in determining prognosis.

The results are consistent with earlier investigations that reported no significant sex-related disparities in MIDCAB outcomes when accounting for baseline differences. Friedrich et al. showed comparable survival and major adverse cardiac and cerebrovascular events-free rates between men and women after MIDCAB, despite higher transfusion rates and longer operative times in women [15]. Gofus et al. also observed no difference in all-cause mortality post-MIDCAB, though wound complications remained more frequent in female patients [5]. Our study extends these findings with a significantly longer follow-up, confirming that minimally invasive revascularization offers durable outcomes in both sexes.

The slightly better survival in matched female patients may be attributable to physiological and procedural factors. MIDCAB avoids cardiopulmonary bypass and sternal division, thereby reducing the risk of hemodilution, transfusion, and sternal wound infections [4]. Women, who are more vulnerable to these complications during conventional CABG due to smaller body surface area and vascular anatomy [5], appear to benefit from the minimally invasive approach. Complete arterial revascularization using the LIMA in all patients likely contributed to sustained graft patency and reduced reintervention [6,7].

Multivariable Cox regression analysis demonstrated distinct predictors of long-term mortality for male and female patients undergoing MIDCAB. In women, advancing age—specifically 70 years or older—was significantly associated with late mortality (HR 2.66, 95% CI 1.02–6.95; *p* = 0.046), consistent with prior reports that underscore age as a key determinant of postoperative outcomes in female cardiovascular patients [4,5]. Cerebrovascular disease also emerged as a powerful predictor of adverse survival in women (HR 5.33, 95% CI 1.49–19.03; *p* = 0.010), aligning with previous studies highlighting the interplay between cerebrovascular vulnerability and coronary pathology [6]. Some counterintuitive hazard ratios in the female subgroup, such as those for age > 80 years, multiple prior myocardial infarctions, and CKD, likely reflect low event counts and statistical instability rather than true protective effects. These findings underscore the importance of cautious interpretation in underpowered subgroups.

Among men, late mortality was influenced by a broader constellation of preoperative and comorbid factors. Age was a significant prognostic variable (*p* = 0.041), and prior cardiac surgery markedly increased risk (HR 4.51; *p* = 0.008). The presence of diabetes (HR 1.86; *p* = 0.042), chronic kidney disease (HR 4.92; *p* = 0.026), and pulmonary disease (HR 2.35; *p* = 0.013) were independently associated with worse survival, reinforcing the cumulative impact of multi-organ dysfunction on surgical resilience [7,8,9]. Cerebrovascular disease remained a strong predictor in men as well (HR 4.77; *p* < 0.001), while preserved LVEF conferred a protective effect (HR 0.17; *p* < 0.001), underscoring the prognostic value of cardiac functional reserve across sexes [10]. While univariate analysis suggested a paradoxical association between poor LVEF and reduced mortality in men, this was not sustained in multivariable modeling and likely reflects statistical noise from small subgroup counts. The protective effect of preserved LVEF in the final model reinforces its prognostic importance.

This study benefits from a large single-center cohort, rigorous statistical matching, and nearly complete long-term follow-up. The reproducibility of data via national audit linkage adds credibility. However, limitations include its retrospective design and the relatively modest female sample, which may underpower detection of subtle differences. Variables such as hormonal status, frailty indices, and intraoperative technical adjustments were not available. The single-institution setting may also limit generalizability to centers with differing procedural volumes or expertise. Data on postoperative myocardial infarction and late percutaneous coronary intervention were not available in the audit-linked dataset, limiting our ability to assess the influence of these events on long-term survival and potential sex-based differences. These outcomes are clinically relevant, as they may reflect differences in graft patency, disease progression, or access to follow-up care. Their absence precludes a more granular understanding of survival trajectories and may obscure important intersex variations in late cardiac morbidity. In addition, the use of 1:1 propensity score matching, while effective in achieving covariate balance, resulted in a reduced matched cohort size. This trade-off was accepted to prioritize internal validity and minimize residual confounding. Alternative approaches such as inverse probability weighting may retain more patients but introduce greater model dependence and heterogeneity.

Clinically, these findings support the safety and efficacy of MIDCAB in women with anatomically appropriate LAD disease—challenging historic assumptions of poorer surgical outcomes among female patients. For policymakers, they underscore the need to expand minimally invasive surgical access, especially for women who remain underrepresented in cardiac surgical registries [17]. These data can inform individualized revascularization decisions and highlight the importance of sex-aware surgical planning.

Future investigations should assess MIDCAB in broader populations, including multivessel disease and ethnically diverse cohorts. Further study of sex-specific perioperative care—such as transfusion thresholds and anemia management strategies—may optimize recovery. Randomized comparisons between MIDCAB and PCI in female patients could determine comparative effectiveness, while biomarker profiling may clarify mechanisms behind sex-related differences in vascular healing and survival.

## 5. Conclusions

In conclusion, MIDCAB offers excellent long-term survival for both sexes, with matched female patients showing promising survival trends. The identification of sex-specific risk factors affirms the importance of personalized perioperative management and advocates for equitable access to minimally invasive cardiac surgical strategies.

## Figures and Tables

**Table 1:** Preoperative demographics.

	Unmatched (*n* = 676)				Matched (*n* = 258)			
Variable	Female (*n* = 138)	Male (*n* = 538)	*p* Value	ASMD	Female (*n* = 129)	Male (*n* = 129)	*p* Value	ASMD
Age	64.6 ± 10.7	63.1 ± 10.5	0.122	0.14	64.1 ± 10.5	63.8 ± 9.6	0.562	0.03
NYHA class			0.011	0.21			0.814	0.06
I	40 (29.0)	179 (33.3)			40 (31.0)	29 (22.5)		
II	52 (37.7)	253 (47.0)			51 (39.5)	71 (55.0)		
III	37 (26.8)	94 (17.5)			31 (24.0)	22 (17.1)		
IV	9 (6.5)	12 (2.2)			7 (5.4)	7 (5.4)		
Congestive HF			0.491	0.07			0.523	0.06
Never	128 (92.8)	507 (94.2)			120 (93.0)	117 (90.7)		
Past	5 (3.6)	22 (4.1)			4 (3.1)	8 (6.2)		
Now	5 (3.6)	9 (1.7)			5 (3.9)	4 (3.1)		
Number of previous MI			0.133	0.15			0.637	0.05
0	92 (66.7)	323 (60.0)			85 (65.9)	82 (63.6)		
1	42 (30.4)	190 (35.3)			41 (31.8)	42 (32.6)		
2	3 (2.2)	9 (1.7)			3 (2.3)	3 (2.3)		
3 or more	1 (0.7)	16 (3.0)			0 (0)	2 (1.6)		
Previous cardiac surgery	3 (2.2)	5 (0.9)	0.228	0.11	2 (1.6)	1 (0.8)	0.561	0.07
Diabetes	41 (29.7)	120 (22.3)	0.068	0.17	35 (27.1)	43 (33.3)	0.278	0.08
Hypercholesterolemia	108 (78.3)	418 (77.7)	0.887	0.01	101 (78.3)	110 (85.3)	0.147	0.09
Hypertension	86 (62.3)	366 (68.0)	0.204	0.12	80 (62.0)	83 (64.3)	0.699	0.05
Smoking			<0.001	0.41			0.864	0.04
Never	78 (56.5)	206 (38.3)			69 (53.5)	69 (53.5)		
Ex-smoker	53 (38.4)	282 (52.4)			53 (41.1)	56 (43.4)		
Current	7 (5.1)	50 (9.3)			7 (5.4)	4 (3.1)		
Chronic RF	2 (1.4)	6 (1.1)	0.746	0.03	2 (1.6)	1 (0.8)	0.761	0.07
COPD/Asthma	10 (7.2)	43 (8.0)	0.771	0.03	10 (7.8)	13 (10.1)	0.512	0.08
Cerebrovascular disease	9 (6.5)	16 (3.0)	0.049	0.17	6 (4.7)	6 (4.7)	1.000	0.00
Peripheral vascular disease	8 (5.8)	23 (4.3)	0.446	0.07	7 (5.4)	4 (3.1)	0.355	0.06
AF	1 (0.7)	24 (4.5)	0.038	0.80	1 (0.8)	2 (1.6)	0.561	0.07
Extent of CAD			0.916	0.02			0.742	0.09
1	76 (55.1)	296 (55.0)			72 (55.8)	78 (60.5)		
2	25 (18.1)	92 (17.1)			24 (18.6)	14 (10.9)		
3	37 (26.8)	150 (27.9)			33 (25.6)	37 (28.7)		
LMS disease	13 (9.4)	45 (8.4)	0.693	0.04	11 (8.5)	9 (7.0)	0.641	0.06
LVEF			0.577	0.08			0.649	0.07
Good (>50%)	120 (87.0)	478 (88.8)			113 (87.6)	111 (86.0)		
Fair (30–50%)	17 (12.3)	49 (9.1)			15 (11.6)	13 (10.1)		
Poor (<30%)	1 (0.7)	11 (2.0)			1 (0.8)	5 (3.9)		
Logistic EuroSCORE	3.3 ± 3.1	3.5 ± 6.5	0.490	0.04	3.2 ± 3.1	3.2 ± 4.4	0.974	0.00

AF = atrial fibrillation; ASMD = absolute standardized mean difference; CAD = coronary artery disease; COPD = chronic obstructive pulmonary disease; LMS = left main stem; LVEF = left ventricular ejection fraction; MI = myocardial infarction; NYHA = New York Heart Association; RF = renal failure.

**Table 2:** In hospital outcomes and short-term mortality.

	Unmatched (*n* = 676)			Matched (*n* = 258)		
Variable	Female (*n* = 138)	Male (*n* = 538)	*p* Value	Female (*n* = 129)	Male (*n* = 129)	*p* Value
Reoperation for bleeding	4 (2.9)	13 (2.4)	0.789	4 (3.1)	3 (2.3)	0.702
Tracheostomy	0 (0)	1 (0.2)	0.612	0 (0)	0 (0)	-
TIA/CVA	3 (2.2)	5 (0.9)	0.228	3 (2.3)	3 (2.3)	1.000
RRT	2 (1.4)	6 (1.1)	0.681	1 (0.8)	2 (1.6)	0.561
Death at 30 days	0 (0)	6 (1.1)	0.213	0 (0)	1 (0.8)	0.316
Mean follow-up (years)	10.9 ± 7.4	10.5 ± 7.2	0.606	10.9 ± 7.4	9.4 ± 6.8	0.104

CVA = cerebrovascular accident; RRT = renal replacement therapy; TIA = transient ischemic attack.

**Table 3:** Comparison of Kaplan–Meier survival for unmatched cohorts.

Time (Years)	Survival (%)	95% CI	Log-Rank *p* Value
Females			0.9729
1	99.27	94.93–99.90	
5	96.51	90.92–98.69	
10	88.80	80.55–93.68	
15	86.95	77.73–92.53	
20	75.56	62.11–84.80	
Males			
1	97.18	95.36–98.29	
5	94.94	92.59–96.56	
10	90.10	86.67–92.68	
15	87.19	83.12–90.34	
20	71.99	64.36–78.26	

**Table 4:** Comparison of Kaplan–Meier survival of matched cohorts.

Time (Years)	Survival (%)	95% CI	Log-Rank *p* Value
Females			0.3370
1	99.22	94.58–99.89	
5	96.26	90.28–98.59	
10	89.30	80.89–94.14	
15	87.32	77.78–92.94	
20	77.57	63.95–86.56	
Males			
1	96.85	91.81–98.81	
5	94.83	88.78–97.66	
10	89.57	81.12–94.37	
15	86.28	76.54–92.18	
20	55.75	33.44–73.23	

**Table 5:** Univariate & Multivariable Cox regression for long-term mortality in females.

Univariate Cox Regression					Multivariable Cox Regression			
Variable	HR	95% CI Lower	95% CI Upper	*p* Value	HR	95% CI Lower	95% CI Upper	*p* Value
Age < 60 years	0.577	0.191	1.744	0.330				
Age 60–69 years	0.846	0.330	2.168	0.727				
Age 70–79 years	2.607	1.000	6.796	0.050	2.658	1.016	6.952	0.046
Age > 80 years	0.566	0.075	4.272	0.581				
NYHA I	1.055	0.389	2.859	0.917				
NYHA II	0.335	0.098	1.152	0.083				
NYHA III	0.668	0.418	1.069	0.092				
NYHA IV	0.889	0.425	1.859	0.754				
No previous MI	1.280	0.800	2.049	0.304				
1 previous MI	0.850	0.522	1.386	0.515				
2 previous MI	4.549	0.000	25,808,102,719.771	0.895				
3 or more previous MI	0.481	0.174	1.334	0.160				
Previous cardiac surgery	3.819	0.865	16.863	0.077				
Diabetes	1.359	0.515	3.586	0.535				
Hypercholesterolaemia	1.653	0.481	5.687	0.425				
Hypertension	1.372	0.540	3.486	0.506				
Never smoker	0.809	0.509	1.286	0.371				
Ex-smoker	1.112	0.703	1.758	0.651				
Current smoker	4.733	0.048	465.553	0.507				
CKD	0.049	0.000	98,483,225.551	0.782				
Pulmonary disease	0.045	0.000	176.983	0.462				
Cerebrovascular disease	5.162	1.456	18.295	0.011	5.325	1.490	19.029	0.010
Peripheral vascular disease	2.287	0.514	10.174	0.277				
Preoperative AF	0.049	0.000	6,695,761,941.549	0.818				
Good EF	1.391	0.748	2.586	0.297				
Moderate EF	0.717	0.386	1.334	0.294				
Poor EF	4.506	0.000	106,883,149,224	0.954				

AF = atrial fibrillation; CKD = chronic kidney disease; EF = ejection fraction; MI = myocardial infarction; NYHA = New York Heart Association.

**Table 6:** Univariate & Multivariable Cox regression for long-term mortality in males.

Univariate Cox Regression					Multivariable Cox Regression			
Variable	HR	95% CI Lower	95% CI Upper	*p* Value	HR	95% CI Lower	95% CI Upper	*p* Value
Age < 60 years	0.384	0.219	0.673	<0.001	0.516	0.273	0.974	0.041
Age 60–69 years	1.119	0.692	1.811	0.646				
Age 70–79 years	2.436	1.465	4.051	<0.001	1.291	0.690	2.418	0.424
Age > 80 years	1.560	0.567	4.293	0.389				
NYHA I	0.663	0.394	1.117	0.123				
NYHA II	1.170	0.730	1.876	0.514				
NYHA III	0.855	0.646	1.131	0.272				
NYHA IV	0.986	0.488	1.993	0.969				
No previous MI	1.065	0.839	1.354	0.604				
1 previous MI	1.053	0.820	1.351	0.686				
2 previous MI	0.608	0.300	1.229	0.166				
3 or more previous MI	0.486	0.306	0.773	0.002	1.626	0.483	5.475	0.432
Previous cardiac surgery	4.618	1.678	12.714	0.003	4.511	1.475	13.796	0.008
Diabetes	1.995	1.154	3.449	0.013	1.858	1.021	3.379	0.042
Hypercholesterolemia	1.408	0.792	2.503	0.244				
Hypertension	1.204	0.739	1.962	0.457				
Never smoker	0.976	0.766	1.245	0.847				
Ex-smoker	0.948	0.748	1.201	0.656				
Current smoker	1.314	0.793	2.178	0.288				
CKD	13.112	4.686	36.694	<0.001	4.922	1.210	20.024	0.026
Pulmonary disease	3.156	1.717	5.802	<0.001	2.349	1.200	4.598	0.013
Cerebrovascular disease	3.883	1.676	8.998	0.002	4.773	1.971	11.556	<0.001
Peripheral vascular disease	2.513	0.996	6.339	0.051				
Preoperative AF	1.871	0.680	5.152	0.255				
Good EF	1.787	1.341	2.382	<0.001	0.167	0.064	0.434	<0.001
Moderate EF	0.646	0.462	0.905	0.011	0.349	0.108	1.134	0.080
Poor EF	0.426	0.270	0.672	<0.001	.			

AF = atrial fibrillation; CKD = chronic kidney disease; EF = ejection fraction; MI = myocardial infarction; NYHA = New York Heart Association.

## Data Availability

The data supporting the findings of this study are available from the corresponding author upon reasonable request.
